# Highly conductive and transparent gallium doped zinc oxide thin films via chemical vapor deposition

**DOI:** 10.1038/s41598-020-57532-7

**Published:** 2020-01-20

**Authors:** Sapna D. Ponja, Sanjayan Sathasivam, Ivan P. Parkin, Claire J. Carmalt

**Affiliations:** Materials Chemistry Centre, Department of Chemistry, University College London, 20 Gordon Street, London, WC1H 0AJ UK

**Keywords:** Chemistry, Materials science

## Abstract

Degenerately doped ZnO is seen as a potential substitute to the ubiquitous and expensive Sn doped In_2_O_3_ as a transparent electrode in optoelectronic devices. Here, highly conductive and transparent Ga doped ZnO thin films were grown via aerosol assisted chemical vapor deposition. The lowest resistivity (7.8 × 10^−4^ Ω.cm) and highest carrier concentration (4.23 × 10^20^ cm^−3^) ever reported for AACVD grown ZnO: Ga was achieved due to using oxygen poor growth conditions enabled by diethylzinc and triethylgallium precursors.

## Introduction

Zinc oxide (ZnO) is a large bandgap semiconducting material with optoelectronic properties that is often used as a transparent conducting oxide (TCO) electrode in photovoltaic devices and flat panel displays^1,2^. TCOs are materials that display both high visible light transparency (>80%) and low electrical resistivity (<10^−3^ Ω.cm). Some of the many advantages of ZnO over the more commonly used tin doped indium oxide (ITO) and fluorine doped tin oxide (FTO) are that it is relatively inexpensive and earth abundant^1,3,4^. Furthermore, ZnO is known to show higher light transmittance and higher resistance to the hydrogen plasma that is often used in the preparation of silicon based photovoltaic devices^5,6^.

ZnO in the pure form is generally too resistive for TCO applications and requires donor dopants such as Al or Ga on Zn sites and/or F on O sites. Such doping results in shallow donor states below the ZnO conduction band minima that are ionized at room temperature to increase carrier concentration and therefore reduce electrical resistivity. The most widely used dopant for ZnO is Al and practical resistivities between 2–3 × 10^−4^ Ω.cm have often been achieved^1,3,4^. However due to the small ionic size of Al^3+^ (0.39 Å) in the four-coordination, it is rather mobile hence leading to stability issues^7^. Also with Al as a dopant, the enthalpy of formation for Al_2_O_3_ is strongly negative (−17.27 eV) and the likelihood of forming electrically inactive Al_2_O_3_ secondary phase is very high^3,8^. Ga_2_O_3_ on the other hand has a less negative formation enthalpy (−11.29 eV) and a larger ionic radius (0.47 Å) compared to Al therefore potentially making it a more stable and efficient donor dopant^3,8^. In literature, ZnO: Ga thin films have been prepared *via* both physical and chemical techniques^9,10^. Gomez *et al*. have shown ZnO: Ga films with 8 × 10^−3^ Ω.cm resistivity by spray pyrolysis deposition on glass^11^. Recently, Szabo *et al*. have shown ZnO: Ga thin films grown using atomic layer deposition on GaN substrates at 300 °C with resistivities in 10^−4^ Ω.cm order^12^. Radio-frequency sputtering was used by Fortunato *et al*. to produced Ga doped films with ~2.8 × 10^−4^ Ω.cm^13^. Gordon *et al*. used atmospheric pressure chemical vapor deposition with diethyl zinc, triethyl gallium and water to synthesise films with the optimal film having a sheet resistance of 3.6 Ω.□^−1^, given that the film was 660 nm thick, this corresponds to a resistivity of ~2.4 × 10^−4^ Ω.cm^6^. Aerosol assisted chemical vapor deposition (AACVD) has also been used to grow ZnO: Ga films with zinc acetylacetonate hydrate as the Zn source and either gallium acetylacetonate^14–17^ or gallium chloride^18^ as the Ga source. All the AACVD grown films had, however limited success due to resistivities typically in the 10^−2^ Ω.cm order^15–18^.

In this paper, we have overcome such issues previously related to ZnO:Ga films grown via AACVD by using, for the first time, diethyl zinc, triethyl gallium and methanol as precursors. The oxygen poor growth conditions allowed by the use of these precursors has enabled, we believe with the support of previous computational studies, the formation of low resistive thin films^19^. The AACVD synthesized films in this study showed ZnO:Ga with 7.9 × 10^−4^ Ω.cm resistivity and >80% visible light transmittance. The sheet resistance was 17.6 Ω.□^−1^, which is comparable to commercially available and widely used TEC 15 (R_sh_ = ~15 Ω.□^−1^) by NSG^20,21^. The achievement, for the first time, of such low resistive ZnO: Ga films *via* AACVD is important as AACVD is an ambient pressure, scalable and highly tunable technique that has industrial importance used for the fabrication of wide variety of thin film materials^22–30^.

## Results and Discussion

Gallium doped ZnO thin films were produced on glass substrates via AACVD at 450 °C by using a toluene solution of ZnEt_2_ and GaEt_3_ with methanol as the oxygen source. The films were highly transparent to visible light and well adhered to the substrate, passing the Scotch tape test and scratch tests by a stainless steel scalpel^31^. The films were resistant to damage from solvents of differing polarity but were damaged by exposure to acid solutions. They were highly stable in air and showed no deterioration in electrical or optical properties.

The concentration of Ga in the films, determined via energy dispersive X-ray spectroscopy (EDS), was 0, 1.0, 5.0, 8.0 at.% when 0, 2.5, 5, 10 mol.% of GaEt_3_ relative to Zn was used in the precursor solution, respectively. This compares well with X-ray photoelectron spectroscopy (XPS) analysis of the films’ surface that showed Ga concentrations to be 0, >1, 5, 10 at.%, therefore suggesting homogenous distribution of the Ga dopant throughout the film with little bulk or surface segregation.

**Figure 1:**
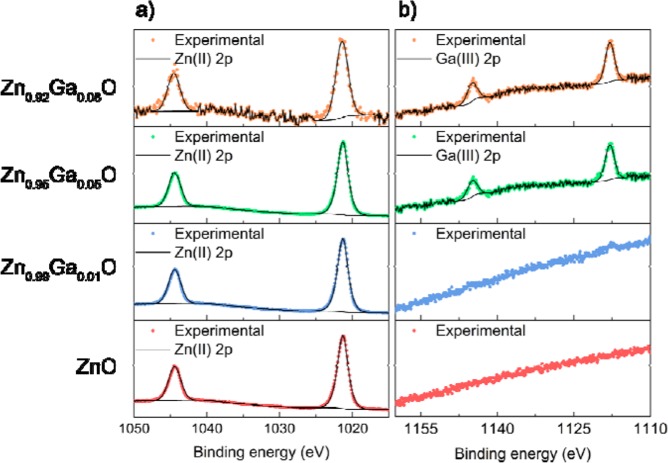
Surface XPS results for the (**a**) Zn and (**b**) Ga 2p transitions for the nominally undoped and ZnO: Ga thin films grown via the AACVD reaction of ZnEt_2_ and GaEt_3_.

Peak fitting of the XPS data for the Zn and Ga 2p transitions was carried out to determine their oxidation states (Fig. 1). The Zn 2p_3/2_ peak was typically centered between 1021.4–1021.5 eV and matching well with literature reports for Zn^2+^ for all films^32^. The Ga 2p peaks for Zn_0.99_Ga_0.01_O was visible (Fig. 1b) but too small to be quantified, however for the Zn_0.95_Ga_0.05_O and Zn_0.92_Ga_0.08_O films, the 2p_3/2_ peak was at 1117.5 eV, corresponding to literature values for Ga^3+^ ^33^.

**Figure 2:**
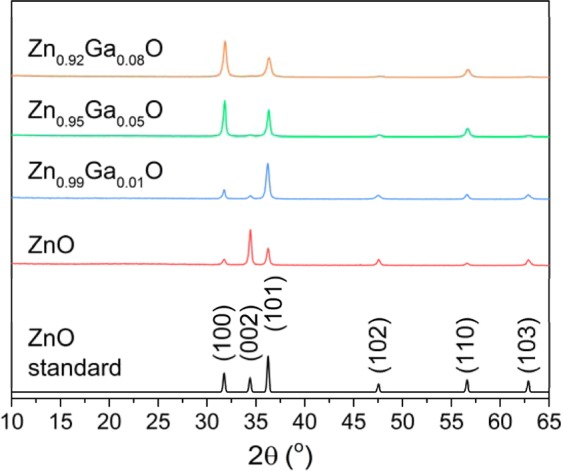
XRD patterns for the nominally undoped and ZnO: Ga AACVD films showing a match to the wurtzite phase of ZnO.

X-ray diffraction (XRD) showed all films matched only the hexagonal wurtzite structure of ZnO (JCPDS 36-1451) (Fig. 2). The Bragg reflections were observed at 31, 34, 36, 47, 56 and 63 °, belonging to the (100), (002), (101), (102), (110) and (103) planes of polycrystalline ZnO^34^. Modeling of these peaks using the Le Bail method showed there was a contraction and a linear decrease in the ZnO unit cell volume as the concentration of Ga increased in the films (Table 1). This gives evidence for successful substitutional doping of Ga into ZnO as the larger four coordinate Zn^2+^ (0.60 Å) is replaced by smaller Ga^3+^ (0.47 Å) ions.

**Table 1:** Variation in the estimated mean crystallite diameter and unit cell parameters of ZnO and GZO thin films with different dopant concentrations of GaEt_3_.

Film	*a*/Å	*c*/Å	Volume/Å^3^	Contraction/%
ZnO	3.2542 (2)	5.2110 (2)	47.791 (5)	—
Zn_0.99_Ga_0.01_O	3.2500 (1)	5.2063 (7)	47.625 (6)	0.34
Zn_0.95_Ga_0.05_O	3.2493 (1)	5.2058 (7)	47.599 (6)	0.40
Zn_0.92_Ga_0.08_O	3.2469 (2)	5.2013 (12)	47.487 (10)	0.64

**Figure 3:**
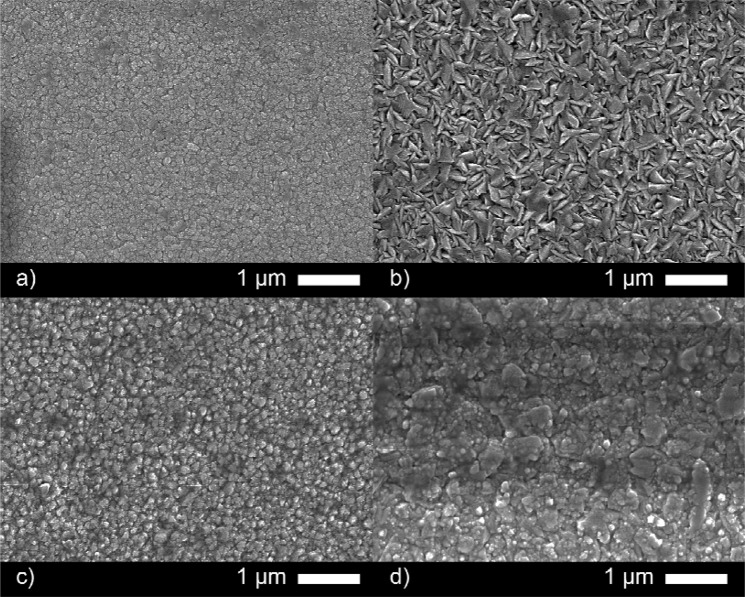
Scanning electron microscopy images of the AACVD grown (**a**) undoped and (**b**) 1%, (**c**) 5% (**d**) 8% Ga doped ZnO films.

The texture coefficient was calculated using the XRD data to determine the degree of preferred orientation in the ZnO films. The nominally undoped film showed a preference for the [002] direction and a lack of growth in the [100], [101] and [110] relative to the standard pattern. Upon doping to 1 at.% with Ga, the XRD pattern showed preference in the [101] direction with growth in the [100] and [002] directions suppressed. This change was also reflected in the scanning electron microscopy (SEM) images (Fig. 3a,b). The morphology of the nominally undoped ZnO film was composed of closely packed rounded clusters ∼100 nm in diameter. In general, the film appeared dense, pinhole free and relatively flat which is somewhat typical of undoped ZnO. At 1 at.% doping the morphology undergoes a dramatic change to show irregular facets (grains) between 300–500 nm wide protruding from the substrate, likely due to growth in the [101] as seen from the XRD. This surface structure has previously been reported for cation doped ZnO systems grown *via* CVD and PVD techniques and is attributed to growth along the low energy *c-*axis direction perpendicular to the substrate that is associated to the [101] or [002] directions^35,36^. This highly textured ZnO is ideal as an electrode for solar cell applications, in particular for amorphous and microcrystalline cells where light scattering and trapping enhances overall efficiency^37^. Further doping to 5 and 8 at.% showed a change back to a flatter morphology with the surface made up of ∼100 nm (5 at.%) and ∼100–500 nm (8 at.%) rounded features protruding only slightly form the substrate (Fig. 3c,d). Texture coefficient calculations for these two films looked similar and showed that preference for the [101] direction had weakened with now a strong preference for the [100] direction.

Side-on SEM imaging was used to determine the film thicknesses (see Supporting Information Fig. S1) to be 400, 400, 450 and 600 nm for the 0, 1, 5, 8 at.% Ga doped films, respectively. These values were used with Hall measurement data to determine the electrical properties of the films.

Hall effect measurements identified all the films as n-type (Table 2). The carrier concentration for the undoped ZnO film was 1.36 × 10^20^ cm^−3^, which is high for an undoped ZnO but not unusual, particularly for films grown from ZnEt_2_, and is generally attributed to adventitious hydrogen^3,35,38,39^. As the concentration of Ga goes up to 1, 5 and 8 at.%, the carrier concentration increases to 2.96 × 10^20^, 4.23 × 10^20^ and 6.55 × 10^20^ cm^−3^ respectively due to the donation of one electron for every Zn^2+^ in the ZnO matrix replaced by Ga^3+^. The doping efficiency was calculated to determine the amount of electrically active Ga in the films by calculating the ratio of the carrier density (minus carrier density in the nominally undoped ZnO) to the Ga concentration. For the 1 at.% film the doping efficiency was 33% but for the higher Ga containing samples the doping efficiency falls to >1%, suggesting much of the Ga in these films is in the form of electrically inactive Ga_2_O_3_ (see Fig. 2) possibly around the ZnO grains (Fig. 3)^40^. This follows, as the enthalpy of formation for Ga_2_O_3_ is quite negative at −11.29 eV compared to that of ZnO (−3.63 eV)^8^. Another likely possibility is self-compensation through acceptor defects, such as Zn vacancies (V_Zn_), and complex donor-acceptor defects, such as Ga_Zn_-V_Zn_ and Ga_Zn_-O_i_^3,19^.

**Table 2:** Summary of the electrical and optical properties of the AACVD grown films. Film thickness: *d*, carrier concentration: n, carrier mobility: µ, resistivity: ρ, sheet resistance: R_sh,_ visible light transmittance: λ_550_, figure of merit from the Haack equation (F.o.M = λ_550_/R_sh_) and optical bandgap: E_opt_.

Film	*d*/nm	*n*/×10^20^ cm^−3^	*µ*/cm^2^ V^−1^ s^−1^	*ρ*/×10^−3^ Ω cm	R_sh_/Ω.sq^−1^	λ_550_/%	F.o.M	E_opt_/eV
ZnO	400	1.36	21.4	2.14	53.6	84	1.6	3.25
Zn_0.99_Ga_0.01_O	400	2.96	15.1	1.40	35.0	86	2.5	3.31
Zn_0.95_Ga_0.05_O	450	4.23	18.7	0.79	17.6	84	4.7	3.52
Zn_0.92_Ga_0.08_O	600	6.55	7.7	1.24	20.7	83	4.0	3.55

This increase in carrier concentration with dopant amount results in a reduction in both the resistivity and carrier mobility (Table 2). The resistivity falls from 2.14 × 10^−3^ Ω.cm for the undoped sample to 1.40 × 10^−3^ Ω.cm and 7.9 × 10^−4^ Ω.cm for the 1 and 5 at.% before rising again to 1.24 × 10^−3^ Ω.cm (8 at.%). The resistivity increases observed for the 8 at.% film despite an increase in carrier concentration, was due to a more than 2-fold drop in the carrier mobility from 18.7 (5 at.%) to 7.7 (8 at.%) cm^2^ V^−1^ s^−1^. This decrease, along with the general reduction in Hall mobility observed for the ZnO and ZnO: Ga films was primarily due to ionized impurity scattering, which is know to be the limiting scattering mechanism for degenerately doped ZnO at carrier concentrations between 10^20^–10^21^ cm^−3^ ^4^.

The sheet resistance – a measure often quoted by TCO manufacturers – was 17.6 Ω.□^−1^ for the film with 5 at.% Ga (Table 2)^20,21^. This represents a 3-fold reduction in sheet resistance compared with the undoped ZnO film and is comparable to commercially available FTO samples (NSG TEC^TM^ 15)^20,21^.

**Figure 4:**
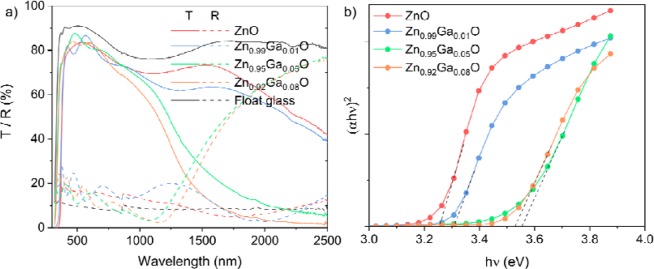
(**a**) UV-vis spectra showing the transmittance (solid lines) and reflectance (dashed lines) across the UV, visible and near infrared wavelengths and (**b**) Tauc plots used to estimate the optical band gaps for the nominally undoped and Ga doped ZnO films on float glass substrates and for the bare float glass substrate.

The optical properties of the thin films were tested across the UV, visible and near infrared (NIR) wavelengths (Fig. 4a). For all films the transmittance to visible light (400–700 nm) was >80%, making them highly suitable for TCO applications. Reflectivity of the films to visible light was low (>20%). For the undoped and 1 at.% Ga film, transmittance across 700–2500 nm remains relatively high. A gradual decrease in transmittance (and corresponding increase in reflectance) was observed indicating that the plasma edge is well into the infrared due to the relatively low (1.36 × 10^20^ and 2.96 × 10^20^ cm^−3^ for 0 and 1 at.%) conduction electron density, similar to what is found in literature^5^. For the 5 and 8 at.% ZnO: Ga films the plasma edge appears at 1445 and 1407 nm, respectively, as a result of their higher carrier concentration. The plasma edge wavelength is an important factor along with the optical band gap in determining the optical window of a TCO material. At wavelengths below the plasma edge and up to the band gap the materials conduction electrons cannot respond and therefore the material is transparent. Above the plasma edge wavelength, the TCO is able to reflect and absorb incident radiation similar to what was observed here.

The optical band gaps of the nominally undoped and ZnO: Ga films were estimated *via* the Tauc plot (Fig. 4b) to be 3.25, 3.31, 3.55 and 3.52 eV for the 0,1, 5, 8 at.% Ga doped films, respectively. The increase in the band gap with carrier concentration follows the expected Moss-Burstein band shift^3,41^.

From the optoelectronic results presented above, it is clear that the low resistivity and optical enhancements of the ZnO films presented in this study are essentially due to increase in carrier concentration as a result of donor doping with Ga^3+^. Compared to the previous four examples of AACVD grown ZnO: Ga found in literature, the resistivites reported here are typically 1-fold and sometimes 2-fold lower (Table 2)^15–18^. This is a consequence of our films having a higher carrier concentration compared to literature at similar Ga at.% in the films. We believe our films have such higher carrier densities due to our synthetic procedure utilizing oxygen poor grown conditions as a result of oxygen free precursors such as ZnEt_2_ and GaEt_3_ with only methanol as the oxygen source. Whereas previous reports have used [Zn(C_5_H_7_O_2_)_2_.H_2_O] and [Ga(C_5_H_7_O_2_)_3_] or GaCl_3_ along with methanol, leading to oxygen rich growth conditions. This theory is supported by computational studies by Demchenko *et al*. on the defect chemistry of ZnO: Ga which showed that the donor-acceptor complex Ga_Zn_-V_Zn_ defect, under oxygen rich growth conditions, has low formation energies compared to the principle donor defect Ga_Zn_, therefore leading to charge compensation and lower carrier densities (x10^19^ cm^−3^ order) in ZnO: Ga^19^. Possibly explaining the poor carrier concentration and resisitivity results seen by Binions *et al*. and Carmalt *et al*. Demchenko’s density functional theory calculations also found that under oxygen poor ZnO: Ga conditions the formation energy of Ga_Zn_-V_Zn_ complex is much higher than that of the donor Ga_Zn_ hence giving rise to high carrier concentrations^19^.

## Conclusion

ZnO: Ga films were prepared by AACVD from the reaction of ZnEt_2_ and GaEt_3_ at 450 °C. The electrical resistivity of the nominally undoped film was 2.14 × 10^−3^ Ω.cm but was reduced to 1.4 × 10^−3^, 7.9 × 10^−4^ and 1.25 × 10^−3^ Ω.cm when Ga concentration was 1, 5 and 8 at.%. The low resistivities observed for ZnO: Ga films here is attributed, possibly, to the O-poor growth conditions that allowed minimization of charge compensation from donor-acceptor complex defects. Optical transparency in the visible wavelengths was above 80% for all the films therefore making the films highly suitable for TCO applications.

## Experimental

### Film deposition

Gallium doped zinc oxide thin films were deposited on SiO_2_ barrier coated float glass. All depositions were carried out under Nitrogen gas (≥99.9% from BOC) at a flow rate of 1.0 L min^−1^ and a temperature of 450 °C. Precursors were purchased from Sigma Aldrich and used as received. The solvents were purchased from Fischer Scientific; toluene was stored under alumina columns and dried with anhydrous engineering equipment and the methanol was dried by distillation over magnesium turnings. The films were deposited using a two-pot AACVD system. An undoped zinc oxide film was produced from one pot containing 0.50 g diethylzinc (15 wt% in toluene) in approx. 20 mL dry toluene and the second pot holding approx. 25 mL dry methanol. ZnO: Ga films were deposited by adding the required mol.% of triethylgallium (2.0 M in toluene) to the diethylzinc and toluene mixture. An aerosol mist of the precursor solutions were created using a piezoelectric device, which was transported to and mixed in a Y-joint before entering the reactor through a baffle. The baffle was water cooled. The depositions lasted between 25–35 minutes. The films were cooled under a flow of nitrogen to room temperature before being removed from the reactor. The films were transparent and colourless and were handled and stored in air.

### Film analysis

The elemental composition in the bulk of the films were calculated using energy dispersive X-ray (EDX) or/and wavelength dispersive X-ray (WDX) spectroscopy. EDX and WDX were carried out using the JEOL JSM-6301F field emission and Phillips ESEM, respectively. The Zn and Ga at.% were obtained from the Zn-Kα line (8638 eV), Ga-Kα (1487 eV) and F-Kα (676.8 eV) X-ray emission lines.

X-ray photoelectron spectroscopy (XPS) was performed using a Thermo Scientific к-Alpha spectrometer fitted with a monochromatic Al-K_α_ (0.834 nm, 1486.6 eV) source. Survey scans were collected in the range 0–1100 eV (binding energy) at a pass energy of 200 eV. Higher resolution scan was recorded for Ga (2p) at a pass energy of 20 eV. For depth profiling, an argon ion gun was used. The peaks were modeled using CasaXPS software and the peak positions were calibrated to carbon (284.5 eV).

X-ray diffraction (XRD) was performed using a modified Bruker-Axs D8 diffractometer with parallel beam optics equipped with a PSD LynxEye silicon strip detector to collect diffracted X-ray photons. This instrument generates X-rays using a Cu source with Cu Kα1 and Cu Kα2 radiation of wavelengths 1.54056 and 1.54439 Å, respectively, emitted with an intensity ratio of 2:1, a voltage of 40 kV, and current of 30 mA. The incident beam angle was kept at 1° and the pattern was collected in the angular range 10 < 2θ < 66° with a step size of 0.05° counted at 4 s/step. The lattice parameters were calculated from X-ray diffraction data using the software GSAS and EXPGUI *via* the Le Bail refinement.

Scanning electron microscopy (SEM) was used to determine the surface morphology and calculate film thickness. The JEOL JSM-6700F and JEOL JSM-6301F Field Emission instruments were used for top down and side on configuration, respectively, at an accelerating voltage of 10 KeV.

UV/Vis/Near IR transmittance and reflectance spectra were taken using the Perkin Elmer Lambda 950 UV-vis/IR spectrometer over a wavelength range of 320–2500 nm with an air background. The band gap was calculated from transmittance and reflectance data using the Tauc plot method.

Hall effect measurements were carried out on an Ecopia HMS-3000 using the Van der Pauw configuration to determine the resistance (ρ), free carrier concentration (*n*) and mobility (μ). Samples of 1 cm^2^ were prepared and silver paint (Agar Scientific) was used to form ohmic contacts. The samples were subjected to an input current of 1 mA and a calibrated magnetic field of 0.58 T.

## Supplementary information

Supporting information.
