# Supplementary material for: Highly conductive and transparent gallium doped zinc oxide thin films via chemical vapor deposition
Source: Sci Rep. 2020 Jan 20;10:638. doi: 10.1038/s41598-020-57532-7 (PMC6971236; doi:10.1038/s41598-020-57532-7)
Supplement: Supplementary file 1 — Supporting information. [file 41598_2020_57532_MOESM1_ESM.doc]

**Highly conductive and transparent gallium doped zinc oxide thin films via chemical vapor deposition**

Sapna D. Ponja, Sanjayan Sathasivam, Ivan P. Parkin and Claire J. Carmalt***

*Corresponding author

*^a^*Materials Chemistry Centre, Department of Chemistry, University College London, 20 Gordon Street, London WC1H 0AJ, UK

E-mail: [c.j.carmalt@ucl.ac.uk](mailto:c.j.carmalt@ucl.ac.uk)


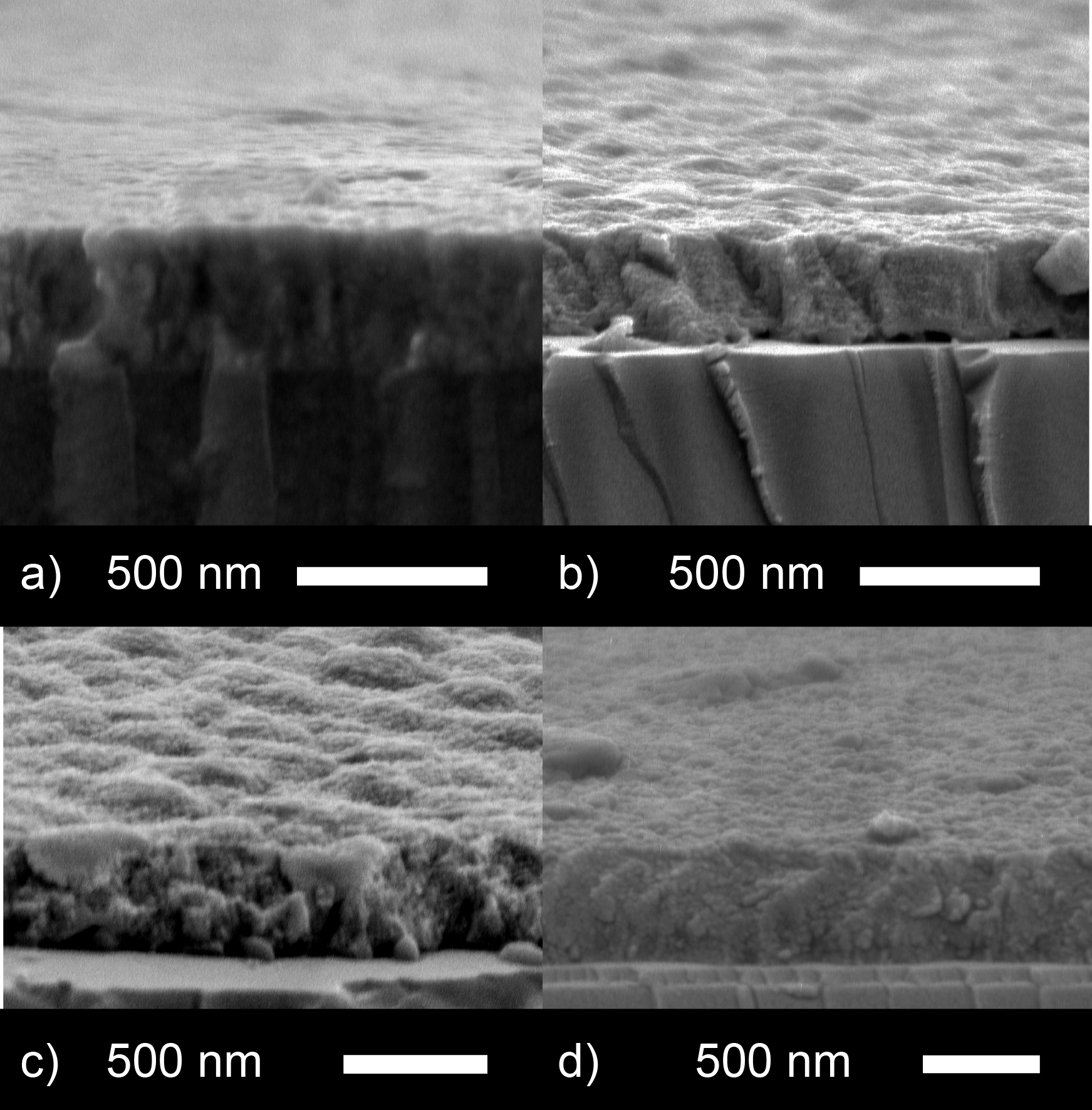


Figure S1: Side-on SEM images showing the thickness of the a) undoped, b) Zn_0.99_Ga_0.01_O, c) Zn_0.95_Ga_0.05_O and d) Zn_0.92_Ga_0.08_O films grown on glass substrates via AACVD.
